# Intrinsic Wettability of Talc

**DOI:** 10.1021/acs.langmuir.5c04929

**Published:** 2026-02-16

**Authors:** Shubhankar Kundu, Lei Li, Haitao Liu

**Affiliations:** † Department of Chemistry, 6614 University of Pittsburgh, 219 Parkman Ave, Pittsburgh, Pennsylvania 15260, United States; ‡ Department of Chemical and Petroleum Engineering, 6614 University of Pittsburgh, 940 Benedum Hall, Pittsburgh, Pennsylvania 15261, United States

## Abstract

Hydrophobicity of talc is traditionally considered as an intrinsic property of this mineral. Here, we show that this hydrophobicity is likely caused by airborne hydrocarbon contamination. We found that a freshly prepared talc surface is much more hydrophilic than previously reported. Upon exposure to ambient air, the water contact angle of talc increases over time. Spectroscopy analysis and control experiments indicate that this wetting transition is due to the deposition of airborne hydrocarbons, the kinetics of which are highly dependent on relative humidity.

## Graphical abstract

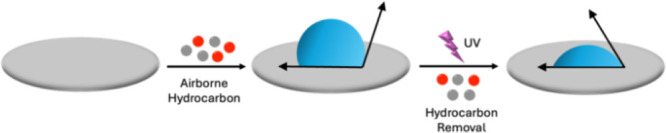

## Introduction

Talc is a naturally occurring clay mineral with a chemical composition of Mg_3_Si_4_O_10_(OH)_2_, belonging to the phyllosilicate family.^1^ It exists in trioctahedral structure and is the softest mineral.^2^ Talc is widely used in cosmetics,^3–5^ paints,^4,6,7^ paper,^4–6,8^ plastics,^4–6^ mining,^9–12^ and rubber^3,4,13^ industries due to its unique properties such as plate-like structure, softness, chemical inertness, and organophilicity.^2^

Talc is a well-known hydrophobic material with reported water contact angles (WCAs) ranging from 80° to 96°.^1,14–17^ However, prior investigations on talc have also reported contradictory results for the wettability of this mineral. Studies on Montana and Vermont talc samples yielded surprisingly low WCAs (around 50°) in sessile drop measurements after treatments with a wheel polisher and ultrasonic cleaning.^18^ Immersion calorimetric studies indicate that outgassed talc is not hydrophobic; in parallel, adsorption measurement of different gases on degassed talc at different temperatures suggests that talc possesses structural microscopic hydrophilicity.^19,20^ Rotenberg et al. modeled water interaction with the talc surface at different environmental conditions to understand the wettability of talc. Their investigation indicates that the wettability of talc surface depends on the relative humidity; at low relative humidity, talc surface behaves as hydrophilic, but at high relative humidity, cohesive interactions between water molecules dominate over adhesive forces between talc and water, making it hydrophobic in nature.^15^ In summary, although talc is generally accepted as hydrophobic, its intrinsic wettability remains a controversy.

Understanding the intrinsic wettability of talc has significant relevance to fundamental research in surface chemistry. Some previous studies on talc have attributed different wettabilities to different facets of its crystal structure. Figure 1 shows the crystal structure of talc with an Mg-based octahedral layer sandwiched between Si rings through shared oxygens via forming siloxane (Si–O–Si) bonds, exposing oxide surfaces held together by weak van der Waals forces.^21^ It has been reported that the basal surface of talc is hydrophobic; the edge surface, consisting of hydroxide ions, Mg, Si, and substituted cations, undergoes hydrolysis upon exposure to water, making it hydrophilic in nature.^1,2,18,21^ Supporting this concept, Yildirim had individually determined acid–base properties of talc from the hydrophobic basal plane and hydrophilic edges.^22^ The dual nature of talc surface has been a well-explored investigation in surface science.^18^

**Figure 1:**
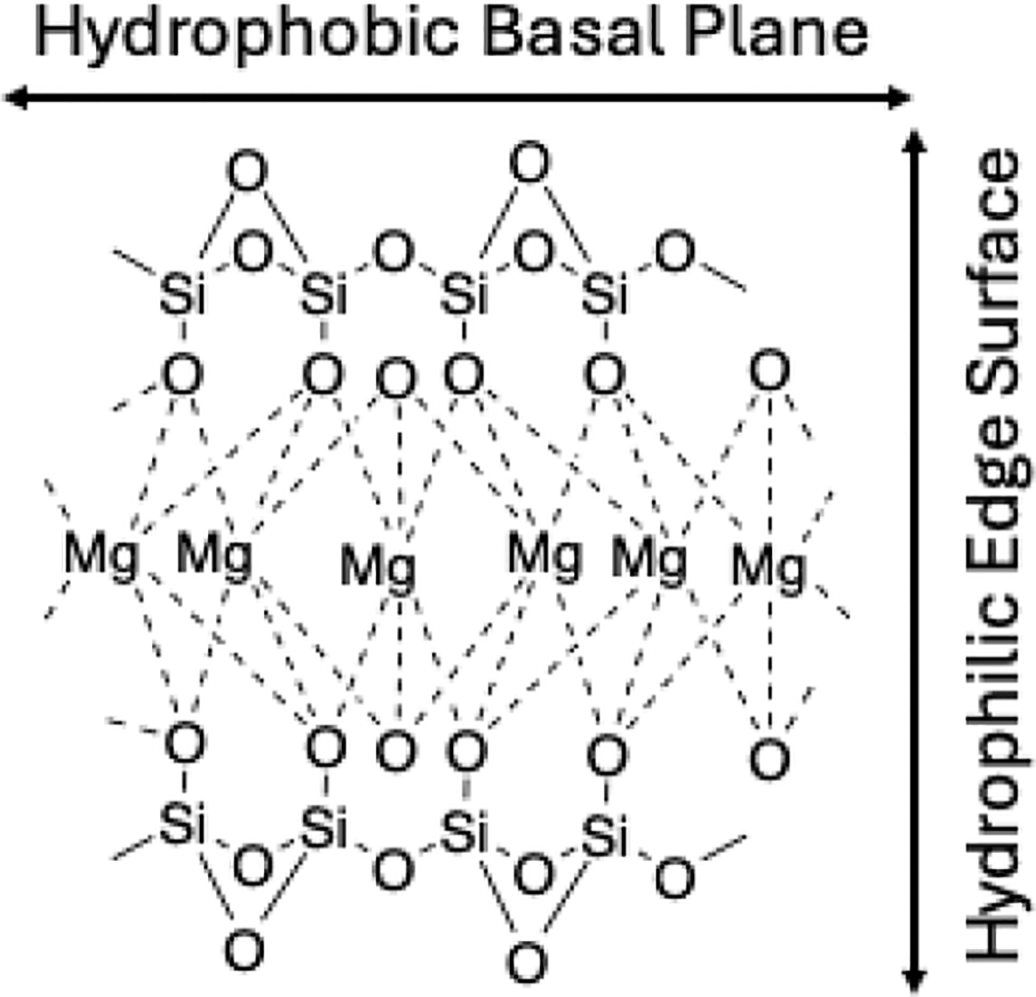
Crystal structure of talc having a hydrophobic basal plane and hydrophilic edge surface.

From a fundamental research perspective, the hydrophobicity of talc is also intriguing because one would have expected hydrophilic behavior from highly polar silicate minerals, such as talc. Previous studies on wetting transition of inorganic materials, such as graphite^23^ and aluminum oxide,^24^ indicate that their inherent hydrophilicity is often masked by airborne hydrocarbon contamination. For example, Li et al. have shown that the WCA of freshly prepared graphitic surfaces increases over time upon exposure to ambient air. Thermal annealing and controlled UV–O_3_ treatment result in complete or partial removal of the contamination, which concurrently decreases the WCA.^23^ Similar behavior has been observed for alumina powder, which is intrinsically hydrophilic in nature (WCA 40° for freshly prepared sample). However, exposure to ambient air elevates the WCA to the range of 146° to 150°, making alumina coating superhydrophobic.^24^ The wettability of rare earth oxide (REO) minerals also remains an open controversy as researchers have reported the airborne hydrocarbons mask the intrinsic nature of the surface, which directly disagrees with the claim of the surface being hydrophobic.^25–29^ Michot et al. in 1994 also proposed the idea that surface contamination may alter the wettability of talc. In their work, they speculated that outgassed talc at 250 °C eliminates superficially adsorbed organic and inorganic species, making it microscopically very hydrophilic.^19^

The wettability of talc is a significant consideration in many industrial settings of its industrial applications. The hydrophobicity of talc is very detrimental to the mining industry,^9–12^ where the floatability of talc creates difficulties in separating talc particles from other valuable sulfide ores during froth flotation.^2,9,13,18,30,31^ Talc is widely used as a filler in the paper, polymer, and cosmetics industries. Understanding its intrinsic properties will be instrumental to model and evaluate the filler–matrix interactions.^32^

This work shows that airborne contamination contributes to the inconsistent wetting data in talc research, analogous to the cases reported for graphene and aluminum oxide surfaces. Specifically, X-ray photoelectron spectroscopy (XPS) revealed the presence of hydrocarbons on the surface; adsorption of airborne hydrocarbons increases the WCA and their removal by UV–O_3_ and/or argon-plasma cleaning restores its hydrophilic nature, as shown in Scheme 1.

**Scheme 1:**
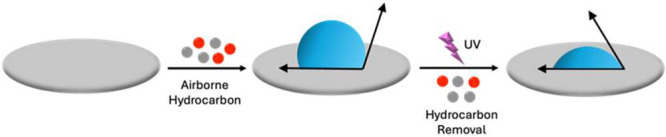
Impact of Airborne Hydrocarbon Contamination on the Wettability of Talc

## Experimental Section

### Materials

The bulk natural talc mineral was purchased from a private vendor (mining location unknown), and its structure was confirmed by X-ray diffraction (Figure S1). Powdered talc was purchased from Thermo Fisher Scientific. Hexadecylamine (98%) and eicosane (99%) were purchased from Sigma-Aldrich. Naphthalene (reagent grade) and anthracene (≥99%) were purchased from Fisher Scientific and Fluka Analytical, respectively. The basal plane of the bulk talc sample was progressively polished by silicon carbide sandpapers (grit size 600, 800, and 1200. *Note: grit size refers to the coarseness of the sandpaper*), with deionized (DI) water rinsing after each round of polishing. After three rounds of polishing and rinsing, the sample was dried with N_2_ and then cleaned by UV–O_3_. Sandpaper treatment was not applied prior to the argon-plasma cleaning of the samples.

### UV–O_3_ Cleaning

A Novascan PSD digital ozone (O_3_) cleaner was used to clean talc samples after being cleaned with sandpaper. The cleaner operates by converting oxygen into ozone and other reactive species by using the high-intensity 185 nm emission from an UV lamp. Simultaneously, the 254 nm UV emission excites organic contaminants to make them susceptible to destruction by the newly formed ozone and other reactive species. The instrument was run under ambient air without using supplemental oxygen for 30 min.

### Argon-Plasma Cleaning

A PDC-001 plasma cleaner purchased from Harrick Plasma was used to clean talc samples under inert conditions, maintaining continuous flow of argon for 15 min at high plasma irradiation. It is possible that UV–O_3_ may introduce oxidants to the surface, making it hydrophilic. Argon-plasma cleaning was used as a control to rule out this possibility.

### WCA Measurements

WCA was measured on the talc sample using a VCA Optima XE instrument via the sessile droplet technique. Each measurement was performed at three different spots on the surface utilizing 1 μL droplets of DI water sourced from a Thermo Scientific Barnstead MicroPure Water Purification System having specification of total organic carbon range 1 to 5 ppb. This procedure was followed for the kinetic study as well. A measurement is taken within 5 s of adding the droplet to the surface. These contact angle measurements were performed in ambient air conditions at ca. 22 °C. Each reported WCA data point is the average value of all the measurements from a similar type of sample. We note that the sessile droplet technique has its own limitations, and a more comprehensive understanding of the wettability can be obtained by the advancing and receding contact angle measurements.^33^

### X-Ray Photoelectron Spectroscopy (XPS) Measurements

XPS data were acquired using an ESCALAB 250Xi XPS instrument, with strict adherence to predefined time intervals throughout the data collection process. Data were collected with a spot size of 650 μm, a photoemission angle of 0° (relative to the sample surface normal), and using monochromatic Al Kα X-ray (*h*ν = 1486.6 eV) at a power of 145 W (10 mA, 14.5 kV). High-resolution XPS data were obtained utilizing a minimum of 15 scans for O 1s and Si 2p and 20 scans for N 1s and C 1s, with a pass energy of 50 eV. The data were processed using Thermo Scientific Avantage software.

## Results and Discussion

### Dependence of WCA on Hydrocarbon Deposition

We found that the wettability of talc is strongly dependent on the local atmospheric environment. Commercially purchased bulk talc mineral (untreated talc) stored in air showed a WCA of 82° (Figure 2A). We then polished the sample with sandpaper, followed by rinsing with DI water and drying with nitrogen flow. Further, this sample was exposed to UV–O_3_ for 30 min. WCA was measured immediately after the UV–O_3_ treatment (treated talc) and was found to be 49° (Figure 2B). Further exposure of the same sample to lab air for 240 h elevates the CA to 81° (Figure 2C).

**Figure 2:**
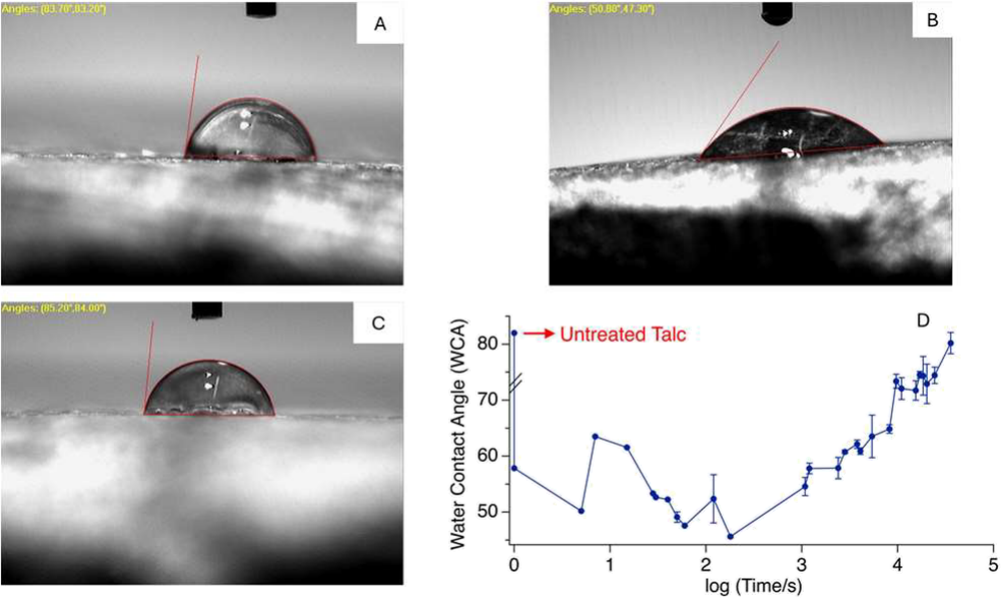
WCA of talc samples (A) before polishing, (B) after polishing and UV–O_3_ cleaning, and (C) after storage in air for 10 days. (D) Semilog plot of WCA (degree) of another talc sample as a function of air exposure (unit of time: seconds).

The air-aged talc sample showed hydrophobic behavior, consistent with many earlier reports.^15,19^ Its WCA is much lower after surface polishing and UV–O_3_ treatment. The reduction of WCA is consistent with what we and others reported for graphitic surface and Al_2_O_3_,^23^ where UV–O_3_ treatment resulted in a reduction of WCA of the surface. This effect is due to the removal of hydrocarbons by reactive oxygen species generated in the UV–O_3_ cleaner.^34^ After exposure to air for 240 h, WCA increased to 81°. This observation is again very similar to what was previously reported for graphitic surfaces and Al_2_O_3_, where surface adsorption of airborne hydrocarbon increases the hydrophobicity of the material. We note that in this case, the kinetics of the wettability transition (10 days) is much slower than in the cases of graphene^23^ and Al_2_O_3_ (e.g., 0.5–2 h).^24^

This hydrophilic-to-hydrophobic transition was consistently observed in multiple experiments. Figure 2D shows another series of WCA data collected from a talc sample over 10 days. Before any kind of surface treatment, the WCA of the sample was 83°. The sample was then polished, treated with UV–O_3_ and stored in a chemistry lab afterwards. The WCA fluctuates between 45° and 65° within 1 h to 5 days after the polishing and UV–O_3_ treatment. Prolonged exposure to air resulted in further elevation in WCA (80° to 85°) within 10 days. We did not know the exact reason behind the large fluctuation of WCA at the beginning of the kinetics study, but we speculate that it may be related to prior exposure to water due to repeated WCA measurements at the same WCA testing location and/or variation in surface roughness. Despite this large fluctuation, the hydrophilic-to-hydrophobic transition is readily reproduced in another three separate experiments.

To investigate whether it is hydrocarbon contamination that influenced the WCA of the polished talc samples, we conducted a positive control experiment, where a freshly polished and UV–O_3_-cleaned talc sample underwent exposure to hexadecyl amine (HDA) vapor at room temperature. Notably, alkyl-amine is a known component of airborne hydrocarbons,^35^ althought the exact chemical nature of the hydrocarbon contaminants is still being studied in our group. In this case, we observed a much faster increase in WCA from 54° (Figure 3B) to 85° (Figure 3C) within a day. Subsequent treatment of the HDA-contaminated talc sample with the UV–O_3_ cleanser restored its hydrophilic nature (WCA: 51°). This result is consistent with the idea that the hydrophobicity of talc is due to surface adsorption of HDA. We have also repeated the experiments to collect finer kinetics data. As shown in Figure 3D, there is a sharp elevation of the WCA within 24 h of exposure to HDA vapor. In comparison, a similar increase in the WCA requires several days of exposure to ambient air (Figure 2D). Similar to using HDA as a model hydrocarbon contaminant, another series of experiments was performed exposing argon-plasma-cleaned talc samples to a mixture of common airborne hydrocarbons like poly aromatic hydrocarbons (naphthalene and anthracene)^36^ and C_20–26_ alkanes (eicosane, C_20_H_42_).^37^ The WCA of plasma-cleaned surface increased from 28 ± 4° to 53 ± 3° upon 24 h of exposure, and the hydrophilicity of the surface was restored once treated with argon plasma again, lowering the WCA back to 32 ± 3°.

**Figure 3:**
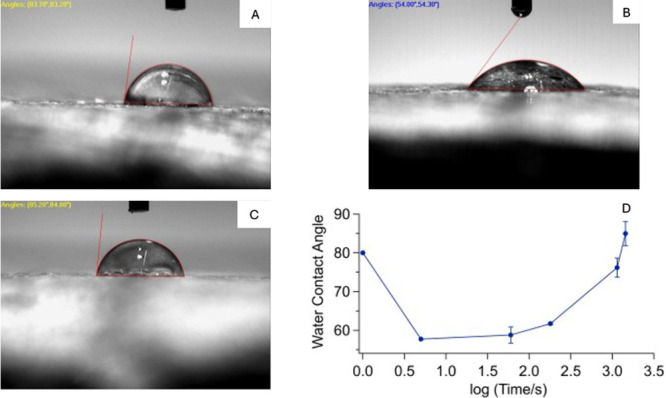
WCA of (A) air-aged, (B) polished and UV/O_3_-treated, and (C) HDA vapor-treated talc sample. (D) Semilog plot of WCA (degree) as a function of exposure time (seconds) to HDA vapor. Data at time zero were taken before polishing and UV/O_3_ treatment.

To further validate the results from bulk talc mineral, we performed similar experiments with commercially purchased talc powder and observed a similar outcome. The talc powder was pressed using a mechanical press into disks (1 cm in diameter) and treated at 500 °C in air. The samples were then exposed to lab air, and their WCAs were measured. Figure 4 illustrates the progression of WCA before heating, after heating, and at 0.16 h (10 min), 24 h, and 240 h of air exposure. The data clearly showed the reduction of WCA after thermal treatment, followed by a slow increase after storage in ambient conditions, mimicking what we observed on bulk samples. We note that this observation is also similar to those reported for Al_2_O_3_ powder^24^ and is consistent with the idea that heating in air oxidatively removes surface hydrocarbon contamination, followed by readsorption of airborne hydrocarbons during storage.

**Figure 4:**
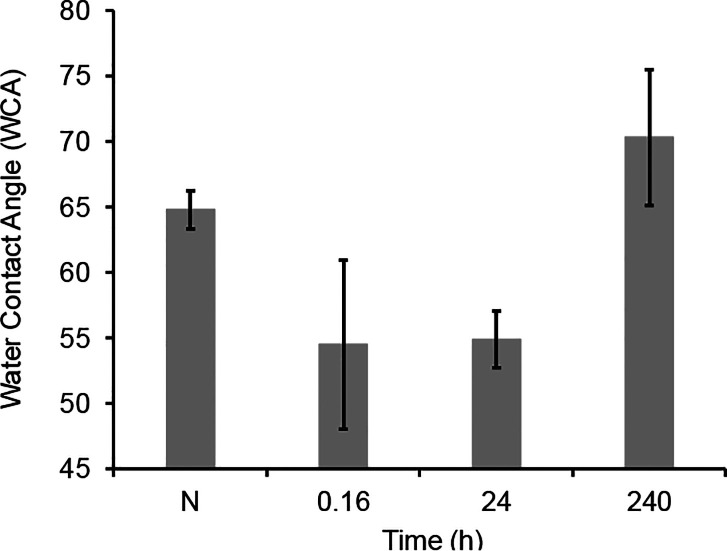
WCA of compressed talc powder before and after heating at 500 °C and air exposure.

XPS provided direct evidence of airborne hydrocarbon accumulation on the talc substrates. Figure 5 compares the C 1s, Mg 1s, and Si 2p XPS spectra derived from the same talc sample: the first data set acquired following a 10 days period of air exposure (untreated talc), the second acquired within 5 to 10 min after removal from the UV–O_3_ cleaning chamber (UV–O_3_-treated talc), and the third after 24 h of HDA exposure (HDA-treated talc). Atomic percentages of C 1s, Mg 2s, Si 2p, and O 1s were calculated from survey spectra for all these three samples, as shown in Table 1. Notably, the percentage of C 1s decreases from 4.0 to 3.1% upon UV–O_3_ treatment and increases eventually to 7.7% when further exposed to HDA, which can be directly correlated to WCAs discussed in later sections.

**Figure 5:**
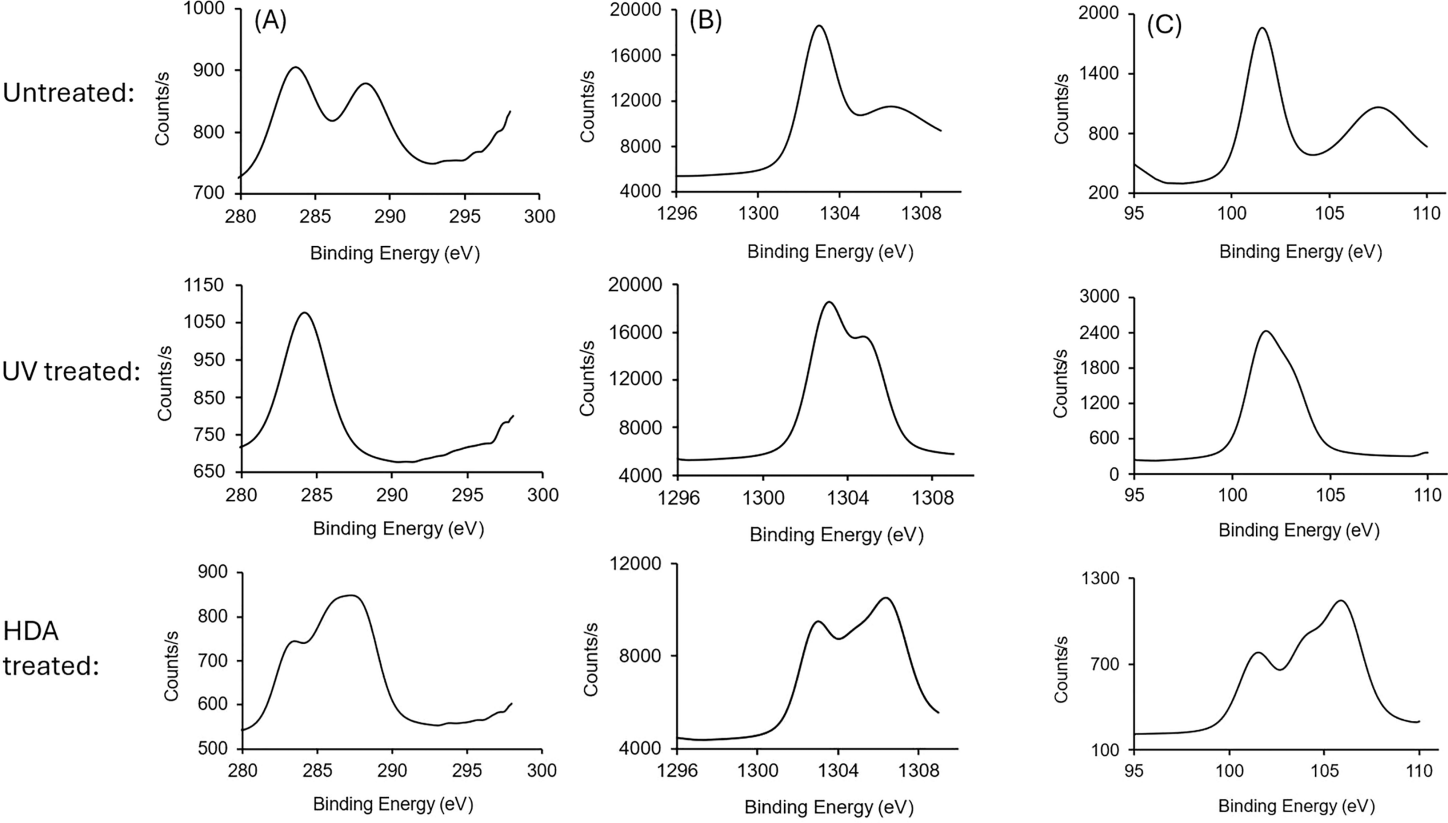
XPS-fitted data for (A) C 1s, (B) Mg 1s, and (C) Si 2p; first row: untreated talc, second row: UV-treated talc, and third row: HDA-treated talc.

**Table 1:** Atomic Percentages Calculated from XPS Survey Spectra

talc sample	C 1s	Mg 1s	Si 2p	O 1s
untreated	4.0	15.5	22.7	57.7
UV treated	3.1	16.6	26.0	54.3
HDA treated	7.7	17.1	23.7	51.5

The C 1s XPS spectrum of the untreated sample showed peaks near 284 and 288.6 eV, which we assign to sp^2^- and sp^3^-hybridized carbon species in the bulk mineral and carbonyl and/or amide species on the surface of the mineral, respectively. The peak at 288.6 eV disappeared after UV–O_3_ treatment, corresponding to airborne hydrocarbon contamination. A new peak reappeared at 286.4 eV after exposure to HDA vapor, corresponding to the C–N or C–O bond. This new peak indicates surface contamination by oxygen- and/or amine-containing hydrocarbons. The same trend can be followed in the case of Mg 1s and Si 2p data, where signals at 1303.2 and 102.1 eV, respectively, do not respond to UV–O_3_ and HDA treatment. In contrast, another set of peaks—Mg 1s at 1307 eV (untreated), 1306.7 eV (HDA-treated) and Si 2p at 108 eV (untreated), 104 eV, 106.1 eV (HDA-treated)—are significantly impacted by the environment. Likely, the first set of peaks is associated with bulk signals, and the second and/or third originate from elements near the surface that are impacted by adsorption/removal of hydrocarbon species. Some shifts in the binding energy of these peaks may be due to the changing nature of the surface species during the experiments (e.g., functional groups present in hydrocarbon contaminants). Throughout the XPS experiments, a strong N 1s peak was not detected; this fact suggests that most HDA adsorbed on talc likely desorbed during XPS measurement. Taken collectively, the XPS data highlight the impact of hydrocarbon adsorption on the chemical environment of talc surfaces.

### Dependence of WCA on Humidity

Previous studies from our group have reported the effect of humidity on the airborne hydrocarbon adsorption on graphite and proposed a mechanism involving competition between adsorption of hydrocarbon vs water vapor in air on graphitic surfaces.^38^ Talc, being another 2D material, shows a similar behavior. To show the effect of humidity on the wetting transition, we conducted the contamination of talc surface by HDA under two conditions. In the first experiment, the talc sample was placed inside a sealed glass container that was maintained at 61% relative humidity with a beaker of water (relative humidity of our laboratory was 49%), and for the other experiment, the talc sample was placed in a dry glass chamber containing calcium sulfate desiccants (relative humidity was 12%).

In both setups, we allowed for the vapor deposition of HDA and conducted time-dependent measurements (0 to 48 h) of WCA. Figure 6 illustrates our data, showing a more rapid hydrophilic-to-hydrophobic transition under dry conditions compared with that under the humid environment. This result is consistent with the idea that adsorption of HDA and other airborne hydrocarbons is slower in a high-humidity environment due to competition with surface-adsorbed water. However, we note that our observation disagrees with the work of Rotenberg et al.*,* which predicted hydrophobic behavior of talc in high humidity environment through molecular dynamics study.^15^

**Figure 6:**
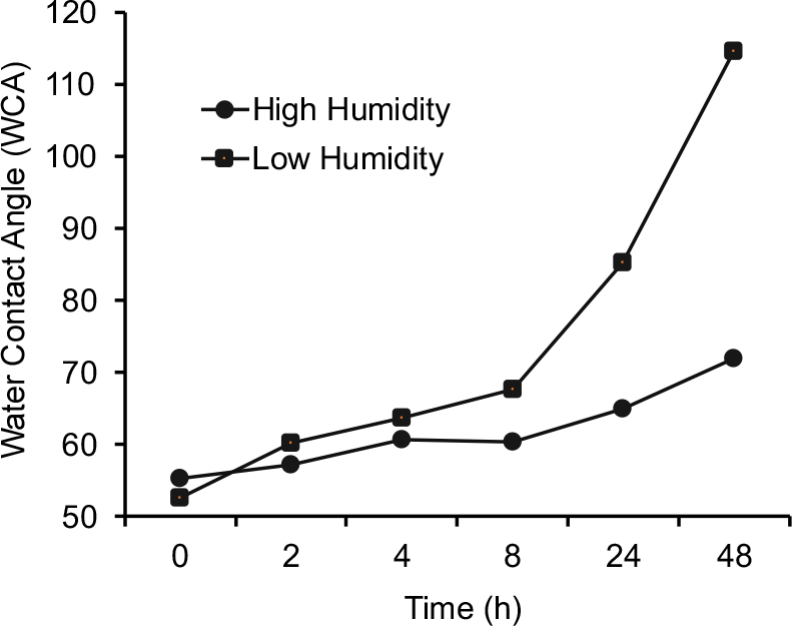
Effect of the humidity on hydrocarbon deposition.

## Conclusions

In this work, we have shown that the hydrophobicity exhibited by talc is a result of airborne hydrocarbon contamination. When polished talc is stored in an ambient environment, we observed an increase in its hydrophobicity within several days. WCA and XPS data attributed the wetting transition to the adsorption of hydrocarbon contaminants from the ambient air. The phenomenon of airborne hydrocarbon adsorption and its impact on WCA has been extensively documented across various surfaces, including gold,^39–41^ aluminum,^42^ graphite,^43,44^ SiO_2_,^43,44^ and TiO_2_.^45^ In all of these cases, airborne hydrocarbon adsorption correlates with WCA elevation similar to what we have observed on talc samples in this work. The timescales of the WCA change exhibit considerable variability, spanning from several minutes (e.g., gold and graphite) to several hours (e.g., SiO_2_ and talc) and are sensitive to additional environmental factors, such as relative humidity. Although talc minerals have one chemical type, their purity and composition can vary depending on their source. So, the results in this work should be interpreted qualitatively or semiquantitatively.

We hope that our finding offers new insights to the wettability of talc. Considering the applications of talc discussed above, these results may have important implications for many industrial practices. Specifically, in processes where the hydrophobicity of talc is relevant (e.g., flotation, additive, coating), we suggest that the underlying mechanism should be revisited, and the material performances are likely affected by organic compounds in the environment.

## Supplementary Material



## Notes

XRD pattern of the talc sample (Figure S1), additional WCA data (Table S1), and additional discussions about XPS data collection (PDF)
