# Supplementary material for: Intrinsic Wettability of Talc
Source: Langmuir. 2026 Feb 16;42(9):6637–42. doi: 10.1021/acs.langmuir.5c04929 (PMC12980818; doi:10.1021/acs.langmuir.5c04929)
Supplement: Supplementary file 1 [file la5c04929_si_001.pdf]

On the Intrinsic Wettability of Talc

Shubhankar Kundu,[a] Lei Li,[b] Haitao Liu*[a]

[a]Department of Chemistry, University of Pittsburgh, 219 Parkman Ave, Pittsburgh, PA, 15260

[b]Department of Chemical and Petroleum Engineering, 940 Benedum Hall, Pittsburgh, PA, 15261

Supporting Information

XRD

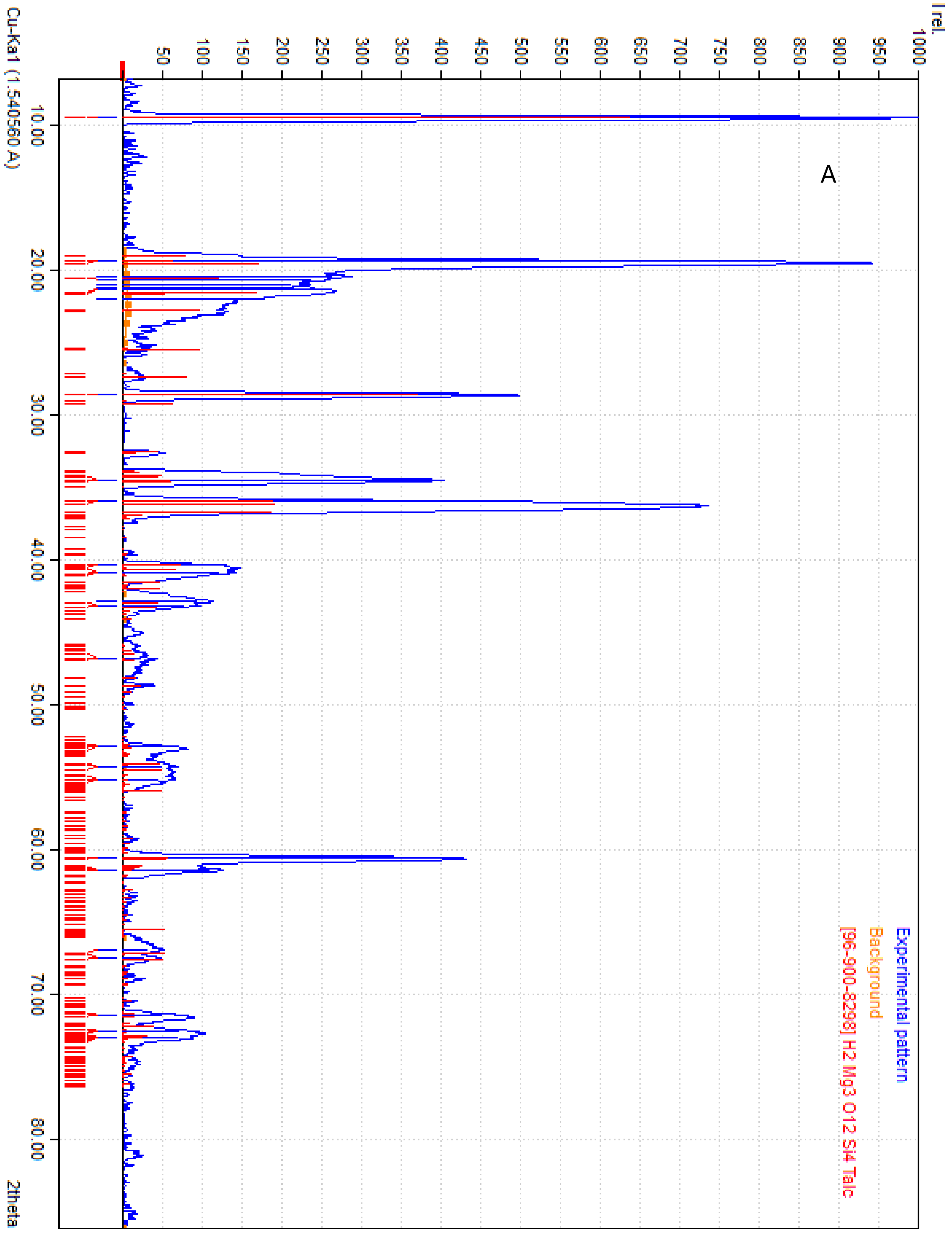
A
I rel.
50
100
150
200
250
300
350
400
450
500
550
600
650
700
750
800
850
900
950
1000
10.00
20.00
30.00
40.00
50.00
60.00
70.00
80.00
2theta
Cu-Ka1 (1.540560 A)
Experimental pattern
Background
[96-900-8298] H2 Mg3 O12 Si4 Talc

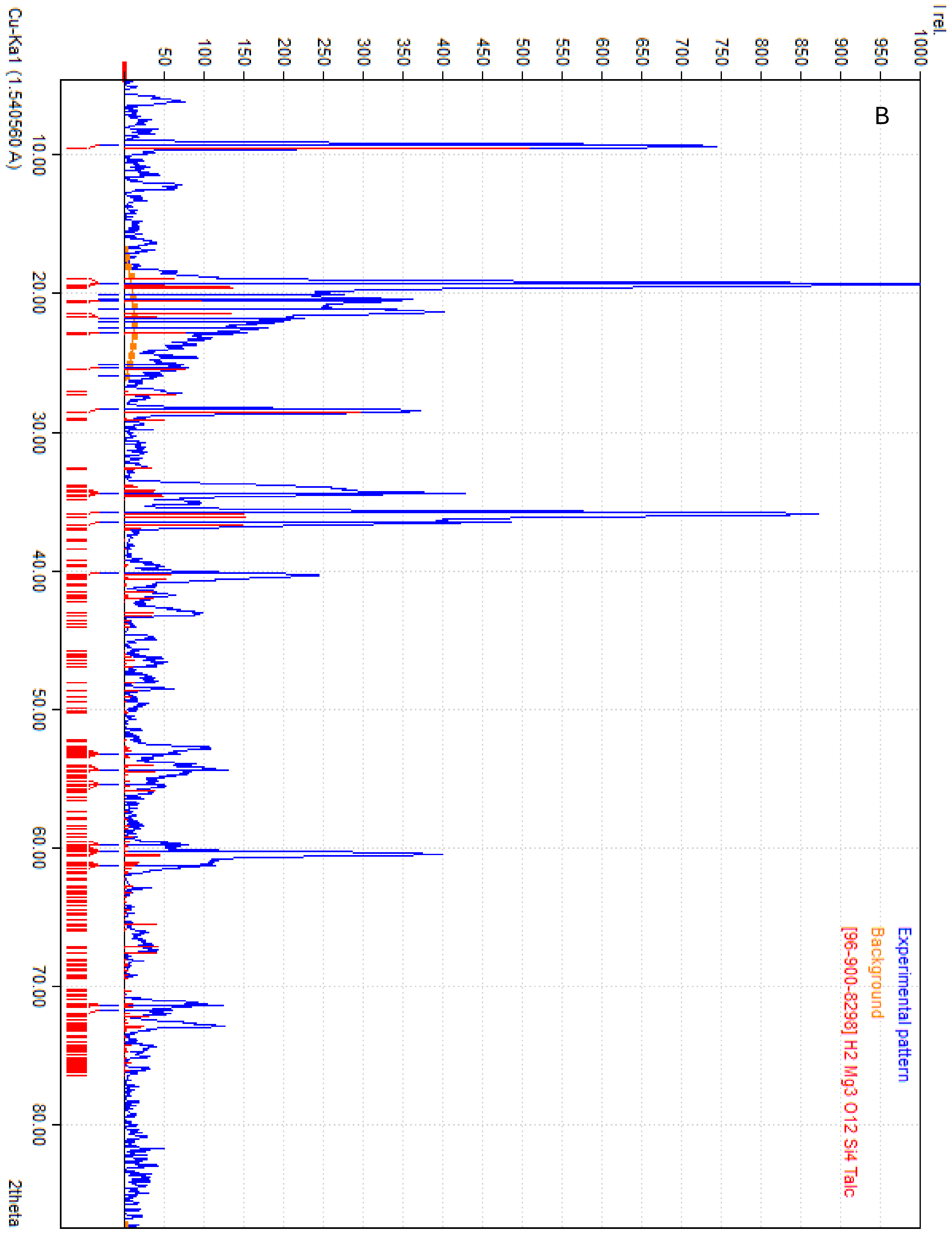

Fig S1: XRD patterns bulk (A) and commercially purchased talc powder (B) samples used in this study. Red lines at the bottom of the plots are reference patterns.

XPS

C1s, O1s, Mg1s, Si2p: These abbreviations correspond to the core-level electrons from the elements detected by the instrument. These signals appear when the electrons are released from the corresponding orbitals (*i.e.*, 1s orbital for C, O, Mg, 2p for Si) upon X-ray irradiation. We are interested in these signals because these elements are the major components of talc.

Extended WCA study

Two additional hydrocarbon models were used in place of hexadecyl amine: 1-hexadecene and a mixture of naphthalene, anthracene, and eicosane (referenced as: common pollutant). **Table S1** shows the change in WCA data before and after exposure to these model compounds for 24 hours, and after plasma cleaning.

Table S1: WCA of the talc samples exposed to other hydrocarbons and air pollutants

Contaminant	Before Exposure (°)	After Exposure (°)	After Ar-Plasma (°)
1-Hexadecene	33±6.2	63±6.3	29±5.4
Common Pollutant	28±3.8	53±3.3	32±2.6
